# Turning terminally differentiated skeletal muscle cells into regenerative progenitors

**DOI:** 10.1038/ncomms8916

**Published:** 2015-08-05

**Authors:** Heng Wang, Sara Lööf, Paula Borg, Gustavo A. Nader, Helen M. Blau, András Simon

**Affiliations:** 1 Department of Cell and Molecular Biology, Karolinska Institute, Solna, Sweden; 2 Department of Physiology and Pharmacology, Karolinska Institute, 17177 Stockholm, Sweden; 3 Baxter Laboratory for Stem Cell Biology, Stanford University, Stanford, California 94305, United States

## Abstract

The ability to repeatedly regenerate limbs during the entire lifespan of an animal is restricted to certain salamander species among vertebrates. This ability involves dedifferentiation of post-mitotic cells into progenitors that in turn form new structures. A long-term enigma has been how injury leads to dedifferentiation. Here we show that skeletal muscle dedifferentiation during newt limb regeneration depends on a programmed cell death response by myofibres. We find that programmed cell death-induced muscle fragmentation produces a population of ‘undead' intermediate cells, which have the capacity to resume proliferation and contribute to muscle regeneration. We demonstrate the derivation of proliferating progeny from differentiated, multinucleated muscle cells by first inducing and subsequently intercepting a programmed cell death response. We conclude that cell survival may be manifested by the production of a dedifferentiated cell with broader potential and that the diversion of a programmed cell death response is an instrument to achieve dedifferentiation.

## Abstract (web summary)

Newts can regenerate amputated limbs via unknown mechanism involving dedifferentiation of cells in the stump into progenitors that contribute to the new appendages. Here the authors show that skeletal muscle dedifferentiation in regenerating newt limbs relies on a diverted programmed cell death response by myofibers.

## Graphical abstract

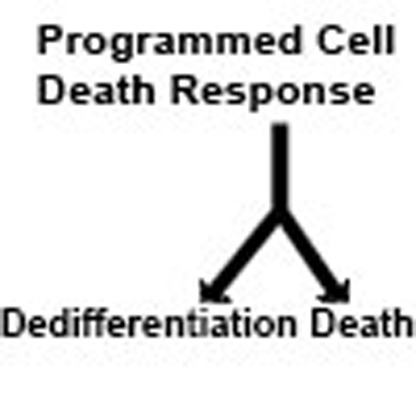

In contrast to mammals, certain salamander species, such as newts, can repeatedly regenerate complex tissues and body parts during their entire lifespan. Regeneration in newts is fuelled by cellular dedifferentiation, which yields cells that constitute an indefinite source of progenitors capable of renewing the lost tissue^1^,^2^,^3^. Two key questions are the mechanisms by which injury leads to dedifferentiation in newts and to what extent such processes are evolutionarily conserved and inducible in mammalian cells. Here we provide clues to both of these questions.

Limb regeneration in newts starts with a rapid wound healing, followed by the formation of a blastema derived from the mesenchyme, which subsequently differentiates into a newly formed fully patterned limb^4^. Blastema formation in newts involves dedifferentiation of myofibres, by which process the multinucleated myofibres fragment into mononucleate cells that in turn downregulate muscle differentiation markers, re-enter the cell cycle and subsequently redifferentiate into myofibres^3^. The underlying mechanisms of myogenic dedifferentiation have remained largely unexplored, and the identity of the stimuli causing the process unknown^5^.

Muscle differentiation can be achieved in tissue culture from proliferating, mononucleate myogenic precursor cells by withdrawal of serum growth factors. As a response to growth factor withdrawal, the mononucleate precursors exit the cell cycle and fuse to each other into a syncytium. The multinucleated myotubes thus formed are the *in vitro* counterparts of myofibres. Although they lack striation and key contractile elements, they are in a stable post-mitotic arrest and express markers of terminal differentiation such as myosin heavy chain (MHC)^6^.

Studies on cultured myotubes showed that compounds causing microtubule depolymerization, such as myoseverin^7^, lead to fragmentation of the syncytium, but rigorous time lapse microscopy analyses demonstrated that the resulting mononucleate cells do not survive to resume proliferation^8^. Other studies indicated that experimentally induced fragmentation of myotubes might lead to proliferating mononucleate cells; however, these studies were typically lacking appropriate lineage-tracing strategies, leaving open the possibility that proliferating cells were derived from pre-existing mononucleate cells in the culture dish^9^,^10^,^11^,^12^.

By combining rigorous fate mapping techniques with molecular manipulations both *in vitro* and *in vivo*, we here show a direct link between myogenic dedifferentiation and programmed cell death (PCD). We find that the multinucleate-to-mononucleate fragmentation is dependent on caspase activity, but the mononucleate cells thus formed re-enter the cell cycle only if the complete execution of PCD is prevented. We also identify that, contrary to newt and mouse C2C12 myotubes, extensive cell cycle re-entry by mononucleate fragments of primary mouse myotubes requires knockdown of p53, reinforcing a key role for appropriate regulation of p53 during limb regeneration.

## Results

### Myotube fragmentation proceeds through a PCD response

**Figure 1:**
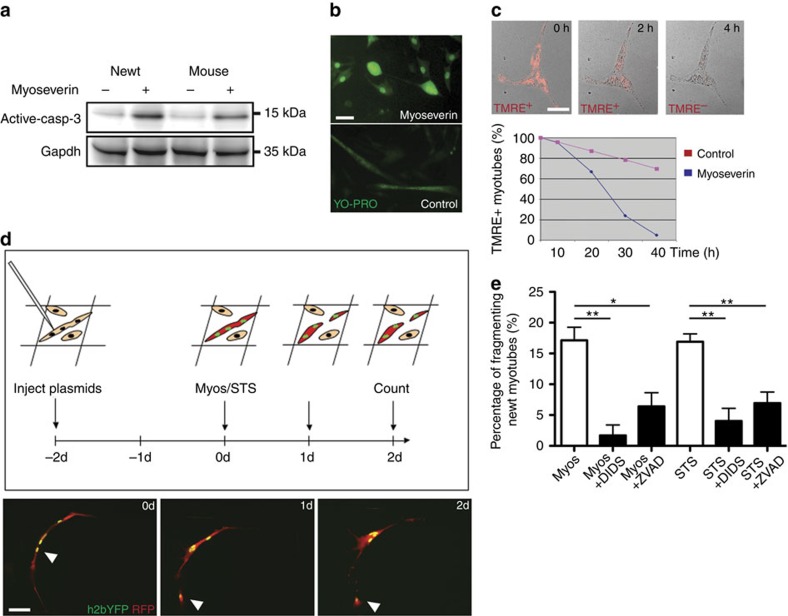
Myotube fragmentation proceeds through a PCD response. (**a**) Western blot analysis showing elevated active-caspase-3 levels in myoseverin-treated (24 h) newt and mouse myotube cultures. (**b**) YO-PRO incorporation in live cultures indicates that fragmenting myotubes are undergoing PCD. Mouse myotubes were treated with myoseverin for 24 h until YO-PRO was added. Scale bar, 10 μm. (**c**) Loss of TMRE in live cultures indicates mitochondrial membrane permeabilization. Example of live imaging of a multinucleated mouse myotube losing TMRE (top). The number of myotubes retaining TMRE in myoseverin-treated compared with control myotubes (bottom). Scale bar, 5 μm. (**d**) Schematic illustration of the cell-tracing strategy of newt myotubes (top). Time lapse images illustrating myotube fragmentation after the STS treatment. Cytoplasmic RFP in red and nucYFP in green (bottom). Scale bar, 20 μm. (**e**) Myoseverin- and STS-induced myotube fragmentation is suppressed by inhibition of mitochondrial membrane permeabilization with DIDS or caspase activity with Z-VAD. Data are represented as mean±s.e.m. (*n*=4 in treatments with Z-VAD, *n*=3 in other treatments, *P<0.05, **P<0.01, one-way analysis of variance (ANOVA)).

The small molecule, myoseverin, has been shown to cause the reversion of the mononucleate-to-multinucleate transition leading to myotube fragmentation^7^; however, rigorous live imaging studies showed that the mononucleate cells derived from myotubes never resumed proliferation^8^. We therefore tested whether myoseverin induced a PCD response by muscle cells. We first examined cultures of myoseverin-treated newt A1 (ref. 13) and mouse C2C12 (ref. 14) myotubes and found that they displayed an increased level of active-caspase-3 compared with controls (Fig. 1a). To directly monitor in live cultures whether myoseverin treatment caused fragmenting myotubes to display a PCD-like phenotype, we employed the nuclear dye YO-PRO, which accumulates in cells undergoing PCD^15^. We found that nuclei in myotubes with fragmenting shape, displaying the typical beads-on-a-string morphology before fragmentation, were YO-PRO^+^ (Fig. 1b). We found that all fragmenting myotubes were YO-PRO^+^ and all intact myotubes were YO-PRO^−^ (Supplementary Table 1). To further examine the myoseverin-induced PCD response, we monitored the integrity of the mitochondrial membrane. Mitochondrial membrane permeabilization is a central event during PCD, and can be monitored in live cells by measuring cellular retention of tetramethyl-rhodamine-ethylester (TMRE)^16^. We found that myoseverin caused rapid release of TMRE, indicating mitochondrial membrane permeabilization (Fig. 1c). These observations together indicated that myoseverin exposure evoked myotube fragmentation and a PCD response without leading to the imminent death of myotubes.

Next, we compared myoseverin and the classical pro-apoptotic molecule staurosporine (STS)^17^ in terms of their effect on myotube fragmentation. This time we combined the treatments with single cell-tracing of myotubes and their progeny, which are produced by fragmentation. To do so, individual newt A1 myotubes were labelled by cellular injection of plasmids encoding cytoplasmic RFP and nucYFP^18^. This technique allowed us to rigorously distinguish between myotube-derived cells from all other cells in the culture dish (Fig. 1d, upper panel). In agreement with earlier tracing experiments^8^, we found that none of the mononucleate progeny that were derived from myoseverin-treated myotubes resumed proliferation, and they eventually died (*n*=45). We further observed that STS caused myotube fragmentation to a similar extent as myoseverin did (Fig. 1e), as illustrated by a time lapse series demonstrating the derivation of a mononucleate cell from a myotube in a STS-treated culture (Fig. 1d, lower panel). Hence, the myotube progeny formed represented long-lived intermediates towards cell death.

Next, we asked whether inhibition of a PCD response interfered with myogenic dedifferentiation. First, we inhibited the voltage-dependent anionic channel, which has been shown to mediate PCD through the mitochondrial pathway. Blocking voltage-dependent anionic channel function with 4,4′diisothicyanatostilbene-2,29-disulfonic-acid (DIDS) prevents cell death after various challenges^19^. We found that both myoseverin- and STS-induced myotube fragmentation was significantly reduced in the presence of DIDS (Fig. 1e). Second, we used the caspase inhibitor Z-VAD, which similarly to DIDS also inhibited fragmentation of myotubes (Fig. 1e). These data showed that the reversal of terminal differentiation is dependent on a PCD response.

### Derivation of cycling progeny by intercepting a PCD response

**Figure 2:**
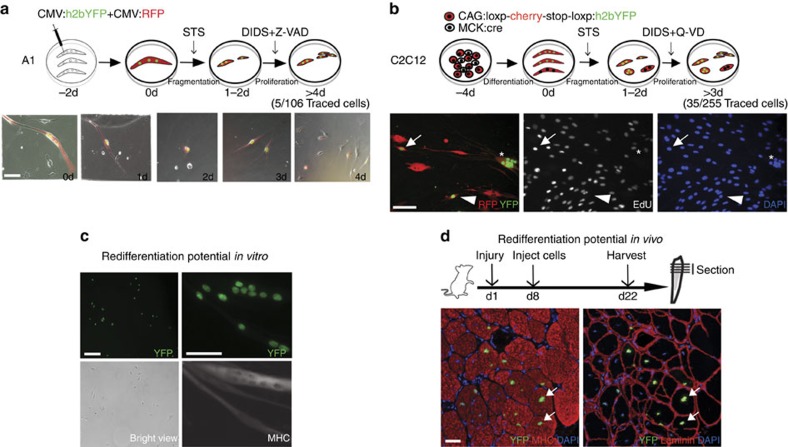
Derivation of the proliferating progeny by intercepting a PCD response. (**a**) Schematic illustration of the treatment and cell-tracing strategies of newt myotubes (top). PCD response was first induced by STS and subsequently inhibited by Z-VAD and DIDS. Live imaging example of a myotube-derived mononucleate progeny (RFP^+^/YFP^+^) that divides (bottom). Scale bar, 20 μm. (**b**) Schematic illustration of the treatment and cell-tracing strategies of mouse C2C12 myotubes (top). PCD response was first induced by STS and subsequently inhibited by Q-VD and DIDS. Cell cycle re-entry indicated by EdU incorporation of a myotube-derived mononucleate progeny (bottom). Arrow points to a YFP^+^/EdU^+^ mononucleate cell. Arrowhead points to a YFP^+^EdU^−^ mononucleate cell. Asterisk points to a YFP^+^EdU^−^ myotube. Scale bar, 20 μm. (**c**) FAC-sorted dedifferentiated C2C12 cells (left) can redifferentiated into myotubes *in vitro* (right). Scale bars, 10 μm. (**d**) On implantation into injured muscle, dedifferentiated cells contribute to myofibre regeneration *in vivo*. Arrows point to centrally localized myonuclei indicative of regenerating myofibre. Scale bar, 20 μm.

The above observations led us to test whether it is possible to derive proliferating progeny from post-mitotic myotubes by first inducing and subsequently inhibiting the PCD response in myotubes. First, A1 myotubes were injected with the lineage tracer and treated with STS to induce a PCD response (Fig. 2a). After 2 days, at the time point when the majority of fragmentation events usually occurred, we blocked mitochondrial membrane permeabilization and caspase activity by DIDS and Z-VAD to rescue cells from further mitochondrial membrane permeabilization-mediated PCD signals and caspase activity. The sequential induction and subsequent inhibition of PCD led to the proliferation of 5 out of 106 individually traced myotube-derived mononucleate cells (Fig. 2a). These dividing myotube-derived cells were morphologically indistinguishable from other proliferating mononucleate precursors in the culture. Given the transient nature of the expression plasmids, we could not trace these mononucleate cells further because they, after resuming proliferation, rapidly lost the fluorescent tracers.

We could, however, circumvent this problem when we tested our PCD-driven dedifferentiation protocol in mammalian C2C12 myotubes. To trace C2C12 myotube-derived cells long term, we developed a genetic recombination strategy. We took advantage of the cell fusion event during myogenic differentiation to achieve expression of a cell tracker specifically in myotubes. As schematically illustrated in Fig. 2b, we generated one line of C2C12 myoblasts transfected with a cherry-to-nucYFP loxP reporter under the ubiquitous CAG promoter, and another line of C2C12 myoblasts, which was transfected with cre-recombinase under the control of the MCK (muscle creatine kinase) promoter. The two lines were mixed together and shifted to differentiation medium, which led to fusion and differentiation of the myoblasts. Since the MCK promoter is only active in differentiated myotubes, and cre-recombinase can only cause conversion of the loxp reporter to nucYFP after fusion with the reporter line, all nucYFP nuclei were found in multinucleate myotubes (Supplementary Movie 1). These myotubes also expressed the late differentiation marker, MHC, and p21 (Supplementary Fig. 1). Some myotubes also showed dual cherry and nucYFP expression when not all copies of the floxed constructs were converted by *cre*. Next, C2C12 myotubes were exposed to STS for 2 days and shifted to medium containing DIDS and the pan-caspase inhibitor Q-VD. Supplementary Movie 2 and Supplementary Fig. 2 show live tracing of a fragmenting myotube until the first division of a mononucleate progeny. We found that out of 255 C2C12 myotube-derived mononucleate cells, 35 resumed proliferation based on incorporation of the nucleotide analogue, 5-ethynyl-2′-deoxyuridine (EdU, Fig. 2b). Proliferating progeny were not obtained without the addition of apoptosis inhibitors after STS treatment (*n*=119). Next, we FACS-purified the YFP^+^ mononucleate cells and examined the expression of the differentiation marker MHC. We found that the majority of the proliferating mononucleate cells already had lost MHC expression (89 out of 105 cells, Supplementary Fig. 3). This finding is in accordance with our previous results gained from *in vivo* fate mapping studies in the salamander limb showing that fragmentation precedes cell cycle re-entry during myogenic dedifferentiation^3^. Thus, similar to salamander A1 myotubes, mouse C2C12 myotubes could also be reprogrammed by first inducing, and subsequently intercepting, a PCD response.

To test the redifferentiation and regeneration potential of C2C12 myotube-derived proliferating cells, we expanded them in culture. We observed that on serum withdrawal, they formed multinucleate myotubes, which expressed MHC (Fig. 2c) and the myonuclei within had exited the cell cycle as assayed by the lack of EdU incorporation (*n*=209). To test the potential of myotube-derived cells in an injury model *in vivo*, we implanted the dedifferentiated cells into bupivacaine-injured tibialis anterior (TA) muscle of nonobese diabetic/severe combined immunodeficiency (NOD-SCID) mice. We observed large areas of regenerating muscle containing YFP^+^ myonuclei 2 weeks following implantation (Fig. 2d). The centrally located myonuclei expressing YFP are indicative that the implanted cells are actively fusing to regenerating myofibres. Thus, dedifferentiated muscle progeny are able to redifferentiate and incorporate into skeletal muscle *in vivo*.

### Inducing proliferation of primary mouse myotube-derived cells

**Figure 3:**
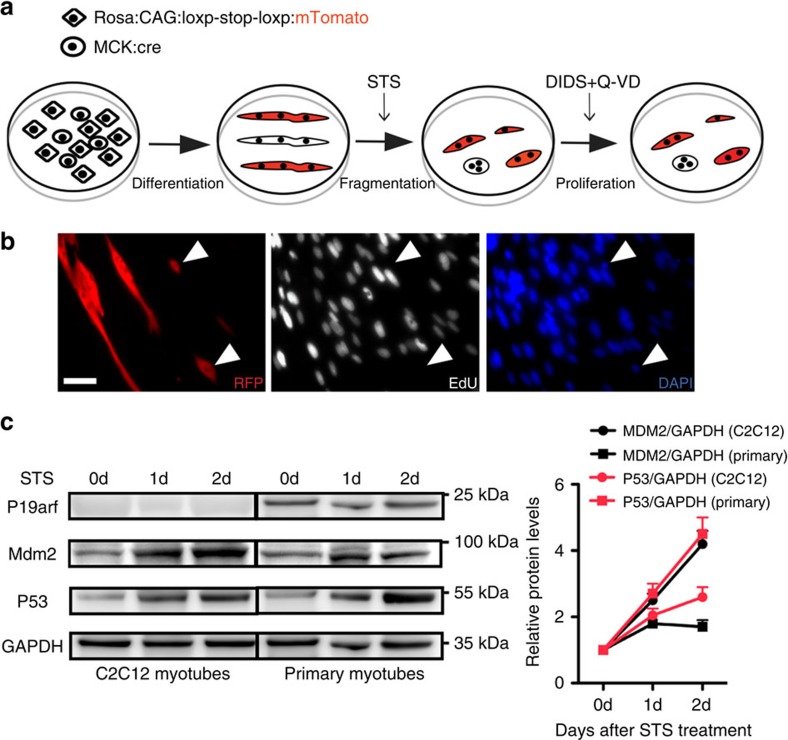
Lack of proliferation in primary myotube-derived mononucleate cells. (**a**) Schematic illustration of the treatment and cell-tracing strategies of primary myotubes. PCD response was first induced by STS and subsequently inhibited by Q-VD and DIDS. (**b**) No primary mouse myotube-derived mononucleate cell was found to re-enter the cell cycle, indicated by the lack of EdU incorporation in RFP^+^ cells (arrowheads). Scale bar, 20 μm. (**c**) Western blot analysis of C2C12 myotube and primary myotube cultures indicates lack of p19 (ARF) expression in C2C12 myotubes. After STS treatment, MDM2 is upregulated less, and p53 is upregulated more in primary myotubes compared with C2C12 myotubes. Date are represented as mean±s.e.m. (*n*=3).

The *p19arf* (alternative reading frame) of the ink4a locus is usually missing in C2C12 cells; hence, we wanted to test the dedifferentiation protocol on primary myotubes formed by the fusion of myoblasts isolated from muscle fibres. In agreement with earlier observations^20^ we found that p19arf was not expressed in C2C12 myotube cultures but was found in the primary myotube cultures (Fig. 3c). To test the dedifferentiation protocol on primary myotubes, we isolated myoblasts from the Rosa26-tomato mice, in which all cells carry a floxed cytoplasmic reporter that becomes expressed upon *cre*-mediated recombination (Fig. 3a). In analogy with the principle described above for tracing C2C12 myotube-derived cells, we let Rosa26 myoblasts fuse with MCK-cre myoblasts, which on fusion into myotubes expressed the cytoplasmic tomato reporter (Fig. 3a). Primary myotube cultures were subjected to the PCD-induced dedifferentiation protocol. Although primary myotubes also underwent fragmentation and gave rise to mononucleate cells, these mononucleate cells, in sharp contrast to the C2C12 cells, did not re-enter the cell cycle as assayed by the lack of EdU incorporation (*n*=435; Fig. 3b).

**Figure 4:**
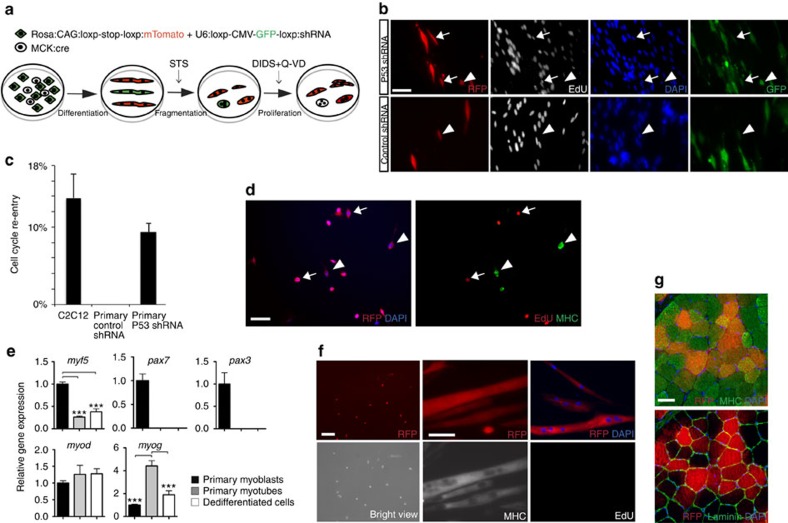
Dedifferentiation of primary myotubes requires p53 knockdown. (**a**) Schematic illustration of treatment, p53 knockdown and cell-tracing strategies in mouse primary myotubes. (**b**) p53 knockdown in myotubes leads to cell cycle re-entry by mononucleate primary myotube-derived progeny, indicated by the appearance of RFP^+^/EdU^+^ cells. Arrows point to EdU^+^ mononucleate cells, arrowheads point to EdU^−^ mononucleate cells. Scale bar, 20 μm. (**c**) Quantification of cell cycle re-entry of mononucleate cells derived from C2C12 myotubes and primary myotubes with and without p53 knockdown. Data are represented as mean±s.e.m. (C2C12: *n*=4, control shRNA: *n*=3, p53 shRNA: *n*=6). (**d**) Sorted primary myotube-derived RFP^+^ cells incorporate the nucleotide analogue, EdU. Arrows point to EdU^+^ mononucleate cells, arrowheads point to EdU− mononucleate cells. Scale bar, 20 μm. (**e**) Real-time PCR analysis of the myogenic transcription factors in primary myoblasts, primary myotubes and proliferating dedifferentiated cells (sorted S and G2/M cells). Data are represented as mean±s.e.m. (*n*=5, ***P<0.001, Student's *t*-test). (**f**) FAC-sorted dedifferentiated mononucleate cells (left) derived from primary mouse myotubes can redifferentiate into myotubes *in vitro* (right). Scale bars, 10 μm. (**g**) On implantation into injured muscle, dedifferentiated cells contribute to myofibre regeneration *in vivo*. Scale bar, 20 μm.

We next analysed the p19arf–p53 axis since it is known that p19arf indirectly stabilizes p53 by causing loss of MDM2, which in turn is a p53 inhibitor^21^. We found that the PCD stimulus by STS led to a differential response in the primary myotubes compared with the C2C12 myotubes in terms of the dynamics of MDM2 expression, showing massive increase in cultures of C2C12 myotube but not in cultures of primary myotubes. In accordance with this observation, we found a robust increase of p53 expression in cultures of primary myotubes but not in cultures of C2C12 myotubes (Fig. 3c). These data indicated that elevated p53 may prevent cell cycle progression in primary myotube-derived cells. To directly test this hypothesis, we wanted to block p53 expression in primary myotubes. Since blocking p53 may interfere with myogenic differentiation^22^, we employed a conditional RNA interference knockdown strategy with a lentiviral vector encoding a short hairpin p53 (Fig. 4a). First, we confirmed that the p53 short hairpin RNA could knock down p53 in primary myotubes (Supplementary Fig. 4). Subsequently, we transduced primary myoblasts from the Rosa26-tomato mice with lentivirus carrying the conditional short hairpin p53 construct, and then co-cultured these myoblasts with MCK-cre myoblasts before differentiation into myotubes. These myotubes thus expressed the cell tracer reporter and were p53-deficient. When we subjected these myotube cultures to our dedifferentiation protocol, we found that mononucleate cells derived from primary myotubes re-entered the cell cycle at similar frequency (9.4±1.1%) as the cells derived from C2C12 myotubes (13.8±3.2%; Fig. 4b,c). In accordance with the observations in the newt limb and in the C2C12 cultures, cell cycle re-entry was a post-fragmentation event since myotubes were not incorporating EdU (*n*=258). Next, we subjected primary myotube-derived cells to FACS based on tomato expression (Supplementary Fig. 5). The vast majority of the mononucleate cells that were incorporating EdU had lost MHC expression (174 out of 177 cells; Fig. 4d). In addition to the loss of MHC, we also examined the expression of myogenic transcription factors using quantitative real-time PCR. We found that *myogenin* was significantly downregulated in dedifferentiated cells compared with the differentiated myotubes. Dedifferentiated cells however retained myod and myf5 expression but did not show expression of pax7 or pax3 (Fig. 4e). On serum withdrawal, the dedifferentiated cells were able to redifferentiate into muscle *in vitro* by forming multinucleate myotubes, which expressed MHC and the myonuclei within had exited the cell cycle as assayed by the lack of EdU incorporation (Fig. 4f). To test the potential of myotube-derived cells in an injury model *in vivo*, we implanted the sorted dedifferentiated cells into the bupivacaine-injured TA muscle of NOD-SCID mice. We observed substantial chimerism in the regenerating area indicated by Tomato^+^ myofibres 2 weeks following implantation (Fig. 4g and Supplementary Fig. 6). These data showed that dedifferentiated myoblasts from primary myotubes are also capable of redifferentiation and fusion with injured myofibres *in vivo*.

### Caspase-dependent dedifferentiation during limb regeneration

**Figure 5:**
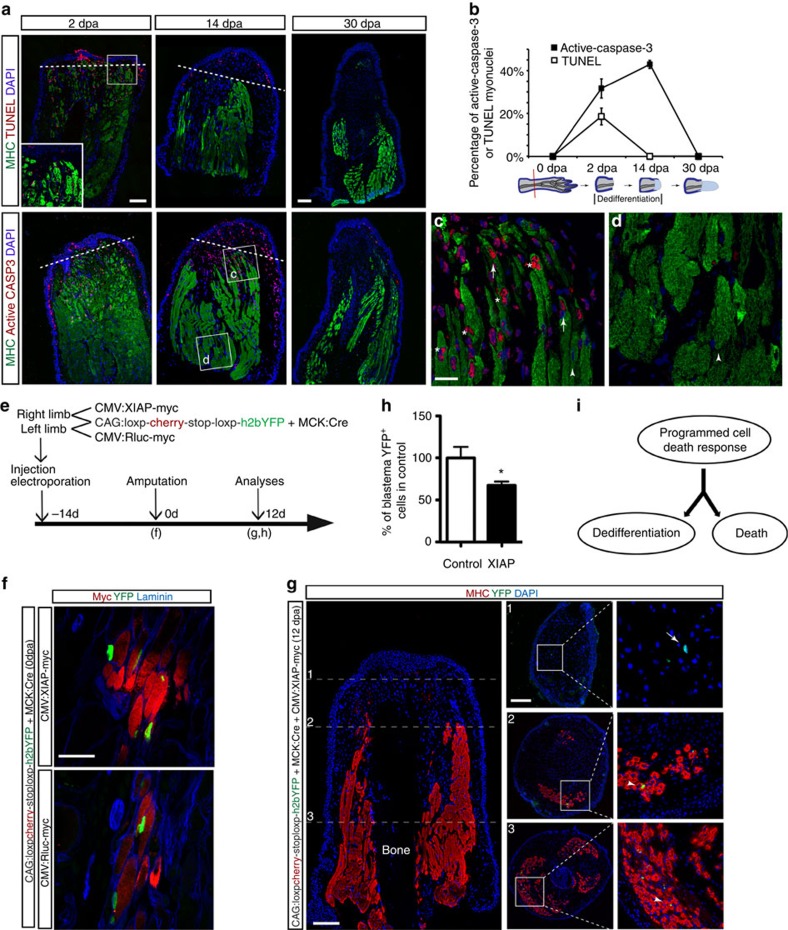
Caspase-dependent myofibre dedifferentiation during limb regeneration. (**a**) TUNEL and active-caspase-3 (red) staining indicate that MHC (green)-expressing myofibres are undergoing PCD. Dashed line indicates the amputation plane. Scale bars, 200 μm. (**b**) Quantification of TUNEL^+^ and active-caspase-3^+^ myonuclei reveals sustained caspase-3 activity but declining number of myonuclei with fragmenting DNA. The right forelimbs were collected from four animals at 0, 2, 14, 30 d.p.a. and sectioned longitudinally. Myonuclei were counted within 2 mm from the tip from three representative sections of each limb. Data are represented as the mean±s.e.m. (*n*=4 limbs in each time point). (**c**,**d**) Active-caspase-3 staining at 14 d.p.a. is more prominent in fragmenting muscle close to the blastema (**c**) compared to proximal (**d**) stump regions. Arrows point to active-caspase-3^+^ myonuclei. Arrowheads point to active-caspase-3^−^ myonuclei. Asterisks mark fragmenting myofibres. Scale bar, 20 μm. (**e**) Schematic illustration of the tracing and caspase inhibition strategies during limb regeneration. (**f**) Illustration of nucYFP (green)-expressing myonuclei within myofibres expressing either the myc-tagged (red) caspase inhibitor, XIAP, or myc-tagged luciferase (red) as detected with an anti-myc antibody. Scale bars, 20 μm. (**g**) Illustration of nucYFP (green)-expressing nuclei in MHC^+^ stump fibres (red) and mononucleate progeny lacking MHC expression (YFP^+^MHC^−^) in blastema. Arrow points to a YFP^+^/MHC^−^ mononucleate cell in the blastema and arrowheads point to YFP^+^ myonuclei in MHC^+^ myofibres in the stump. Scale bars, 200 μm. (**h**) Inhibition of caspase activity by XIAP leads to reduced number of dedifferentiated myofibre progeny in the blastema. Data are represented as mean±s.e.m. (*n*=7 limbs in each treatment, *P<0.05, Student's *t*-test). (**i**) Model illustrating that if the full execution of programmed cell death is prevented, cell survival can be manifested in the production of a dedifferentiated cell.

To test the role of PCD during myogenic dedifferentiation in a *bona fide* regeneration context, we analysed newt limbs following amputation. First, we performed TdT-mediated dUTP nick end labeling (TUNEL) staining, which identifies cells in the late stage of PCD when the DNA is undergoing fragmentation^23^. As shown in Fig. 5a, we found a substantial number of TUNEL^+^ myofibres 2 days post amputation (d.p.a.). The fraction of TUNEL^+^ myonuclei rapidly decreased from 2 d.p.a. to essentially no TUNEL^+^ myofibres at 14 d.p.a. (Fig. 5b).

To corroborate the PCD-like phenotype of myofibres, we performed immunostaining for active-caspase-3. We found that, similar to TUNEL staining, a large number of myofibres were active-caspase-3^+^ at 2 d.p.a. (Fig. 5a). However, we found a difference between the temporal dynamics of DNA fragmentation and caspase-3 activation. In contrast to TUNEL staining, a substantial fraction of myofibres remained active-caspase-3^+^ during the entire dedifferentiation phase of limb regeneration, during which stage skeletal muscle gives rises to undifferentiated blastema cells^24^ (Fig. 5b–d). In addition, the fraction of active-caspase-3^+^ myonuclei exceeded the fraction of TUNEL^+^ myonuclei (Fig. 5b), indicating that not all active-caspase-3^+^ myonuclei have fragmented DNA. Both at 2 and 14 d.p.a. we found cells that were positive for active-caspase-3 but negative for TUNEL (Supplementary Fig. 7). These temporal differences in active-caspase-3 and TUNEL stainings are consistent with the results on cultured myotubes and suggest that activation of caspase-3 in dedifferentiating muscle cells does not always lead to full execution of PCD. To validate the immunostaining for active caspase-3, we injected the fluorescent dye, FAM-VAD-FMK (FLIVO), which is a pan-caspase substrate; thus, it is an independent indicator of caspase activity^25^. We found excellent correlation between the active-caspase-3 immunostaining and the FLIVO signals in regenerating limbs (Supplementary Fig. 8). In agreement with the above observations, we also found strong FLIVO signal specifically in skeletal muscle after amputation (Supplementary Fig. 8).

To further characterize the dedifferentiation stage of limb regeneration with respect to caspases, we analysed the mRNA expression of several genes encoding proteins with key functions in PCD. We found significant and persistent increased expression of several caspases after injury. In contrast, the expression level of the caspase inhibitor, *XIAP* (X-linked inhibitor of apoptosis protein), was reduced early after injury (Supplementary Fig. 9).

These results indicated that caspase activity may be required for myofibre dedifferentiation. To directly test this hypothesis, we determined whether XIAP overexpression would inhibit caspase activity and myofibre dedifferentiation. First, we cloned XIAP from newts (*nXIAP*) and tested its functionality *in vitro*, in A1 cells. We found that *nXIAP* expression inhibits caspase activity in cells undergoing STS-induced PCD (Supplementary Fig. 10). We also transfected limbs with either *nXIAP* or control plasmid and determined the fraction of FLIVO^+^ cells after amputation. These experiments showed a fourfold decrease in the fraction of FLIVO^+^ cells transfected with *nXIAP* compared with control-transfected cells (Supplementary Fig. 10), demonstrating that *nXIAP* inhibited caspase activity.

Next, we used *nXIAP* overexpression in cell-tracking experiments to assess the effect of caspase inhibition on myofibre dedifferentiation *in vivo*. As schematically illustrated in Fig. 5e, we overexpressed myc-tagged *nXIAP* in one limb and myc-tagged luciferase as control in the contralateral limb. Both limbs were simultaneously electroporated with cell-tracking constructs that allow assessing the number of mononucleate cells derived from dedifferentiating multinucleate myofibres, as we previously reported^3^. The cell-tracking constructs were composed of a floxed Cherry-to-nuclear (nuc) YFP reporter and a *Cre*-recombinase under the control of the muscle-specific MCK promoter. Since recombination only occurs in syncytial myonuclei, all mononucleate nucYFP^+^ blastema cells will be derived from myofibres, as previously demonstrated^3^. Accordingly, transfected YFP^+^ myonuclei were found in myc^+^ myofibres (Fig. 5f), and YFP^+^ nuclei were present exclusively in myofibres expressing the late differentiation marker, MHC as previously demonstrated^3^. Following amputation, we determined the extent of myofibre dedifferentiation by counting mononucleate cells that are nucYFP^+^ but had lost MHC expression. We found that the dedifferentiated YFP^+^ cells were significantly reduced in the blastema of those limbs, which were transfected with *nXIAP* compared with limbs, which were transfected with control plasmid (Fig. 5g,h). YFP-labelled myofibres always expressed the XIAP construct, and the vast majority of the transfected constructs were targeting muscle suggesting, in accordance with the culture-based assays, that the inhibition was a cell-autonomous process. The above results collectively demonstrate that myofibres in the stump display features of cells undergoing PCD during the dedifferentiation phase of limb regeneration and that myofibre dedifferentiation is dependent on caspase activity.

## Discussion

In this work we established a link between PCD and myogenic dedifferentiation, and demonstrated that the two processes share common features. We showed that an intercepted PCD response brings about the production of proliferating progeny from terminally differentiated multinucleate muscle cells and that cell survival can be manifested in the birth of a dedifferentiated cell. Several studies identified that until a point of no return cells can be rescued, leading to survival and to the restoration of the original phenotype^26^. On the basis of our results we propose that after embarking on a death programme cells may have more than just a binary decision option between death and returning to the original phenotype. Instead, the point of no return could also represent a junction where cells are susceptible to be redirected towards alternative fates (Fig. 5i).

It has been proposed that caspases can exhibit vital functions and their pro-death role was acquired later during evolution^27^. Indeed, several sets of experiments have indicated that caspase activation is important for cellular differentiation^28^ and regeneration of body parts^29^,^30^,^31^. However, these studies suggested that apoptotic cells promote a compensatory proliferative response in neighbouring cells. While such a process may also be active during regeneration of the newt limb, a complementary model based on our results is that an intercepted PCD response leads to muscle dedifferentiation in a cell-autonomous manner. In accordance with this model, it has been shown that minor injury to the muscle fibres is required to produce mononucleate blastema cells from myofibres during salamander tail regeneration^32^. However, the quantitative relationship between compensatory proliferation by reserve cells and cell-autonomous dedifferentiation needs to be further investigated. It also remains to be determined how comparable dedifferentiation by contractile mammalian myofibres could be evoked.

Our results, on one hand, contrast with an earlier conclusion claiming that pro-apoptotic stimulus does not lead to myogenic dedifferentiation^33^. It is noteworthy that this conclusion was based on a methodology, which did not involve the sequential pro- and anti-apoptotic protocols that we have developed in the present work. On the other hand, our results are in line with several other previous studies on cellular reprogramming and limb regeneration. First, caspases have been implicated in playing a role in the induction of human pluripotent cells^34^. Second, we found that primary mammalian muscle cells require, in addition to the apoptotic stimulus, knockdown of p53 to give rise to proliferating progeny. This is in agreement with studies showing that p53 is activated to suppress the production of induced pluripotent cells^35^,^36^,^37^. Third, it has also been shown that p53 activity is temporally reduced during the early stages of salamander limb regeneration^38^. A recent study on salamander myotubes showed that p53 downregulation is mediated by sustained ERK activation during the return to the cell cycle from the post-mitotic state^39^, raising the question of how ERK activation relates to the apoptotic response in salamander and in mammalian muscle cells. The tumour suppressor p53 has also been shown to play a critical role in regulating the fate of neoblasts during planarian regeneration^40^, placing p53 into the critical interface between tumour and regeneration biology^41^. Our comparative analyses on newt and two different types of mammalian myotubes extends the view that p53 acts as a barrier during dedifferentiation, indicating that the evolution of arf of the ink4 locus in mammals^20^,^42^ could be responsible for the diminution of dedifferentiation capability.

## Methods

### Antibodies and reagents

The following primary antibodies were used: anti-MHC (MF20, Developmental Studies Hybridoma Bank, 1:1,000), anti-active-caspase 3 (ab13847, Abcam, 1:500), anti-cleaved caspase 3 (9661, Cell Signaling, 1:500), anti-YFP (ab6673; Abcam, 1:1,000), anti-RFP (600-401-379, Rockland, 1:500), anti-myc (05-724; Millipore, 1:100), anti-P53 (sc-99, sc-6243, Santa Cruz, 1:200), anti-MDM2 (sc-965, Santa Cruz, 1:200), anti-P19 (ab80, abcam, 1:500) and anti-GAPDH (AM4300, Invitrogen, 1:1,000). Appropriate horseradish peroxidase-conjugated and Alexa Fluor-conjugated secondary antibodies were used for western blot and immunofluorescence studies. The following reagents were used: Staurosporine (BD Biosciences) was used in concentrations ranging between 0.005 and 1 μM. Myoseverin (Sigma) was used at 25 μM. 4,4′ diisothiocyanatostilbene-2,2′-disulphonic acid (Molecular Probes) was used at 100 μM. TMRE (Invitrogen) was used at 10 nM. Z-VAD (Santa Cruz) or Q-VD-OPH (Santa Cruz) was used at 10 μM. YO-PRO-1 (Molecular Probes) was used at 1 mM. FLIVO tracer was from ImmunoChemistry Technologies. TUNEL assay was performed with *In Situ* Cell Death Detection Kit (Roche). EdU staining was performed by incubating 30 min with 100 mM Tris, 1 mM CuSO_4_, 10 μM fluorescent azide and 100 mM ascorbic acid^43^.

### Animals and procedures

Adult red-spotted newts, *Notophthalmus viridescens*, were supplied by Charles D. Sullivan Co. (Nashville, TN, USA). Animals were anaesthetized by placing them in an aqueous solution of 0.1% ethyl 3-aminobenzoate methanesulfonate salt (Sigma) for 15 min. Upper forelimbs were amputated by cutting ∼2–3 mm above the elbow, and the bone and soft tissue were trimmed to produce a flat amputation surface. Animals were left to recover overnight in an aqueous solution of 0.5% sulfamerazine (Sigma). At specified time points, the regenerating limbs were collected from the utmost shoulder of the animal after anaesthetization. FLIVO probe (6 μg per animal) was injected into the pulmonary artery 1 h before killing.

For mouse muscle regeneration experiment, a total volume of 30 μl of 0.75% bupivacaine was injected into the TA muscle of 2-month-old NOD-SCID female mice under anaesthesia (Day 1). The dedifferentiated cells (10^5^ h2bYFP^+^ C2C12 cells or 5 × 10^4^ tomato^+^ primary cells in 30 μl PBS) were implanted into the same muscle (Day 8). The TA muscle was harvested on Day 22. All animal experiments were performed according to the European Community and local ethics committee guidelines.

### Plasmid injection and electroporation

*Schematics of the DNA constructs.* The BIR domains (Baculovirus Inhibitor of Aproptosis Protein Repeats) from XIAP were cloned downstream of a constitutive promoter (cytomegalovirus (CMV)) and fused to a Myc tag. The RING domain was omitted from the construct because previous work^44^ had demonstrated that its inclusion leads to proteasomal degradation of the XIAP protein. Thus, the three BIR domains alone were used to inhibit caspase-3-mediated apoptosis; without concern the effect would be shut down via a negative feedback loop. The Renilla luciferase (Rluc) gene was cloned into the same plasmid backbone as control (Supplementary Fig. 10). Plasmids were purified using the high-purity maxiprep system (OriGene) and were resuspended in 10 mM Tris HCl (pH 8.5) at 10 μg μl^−1^. The different plasmids were mixed with a molar ratio of 1:1:1, which gives the most efficient transgene expression. Plasmid solution (2 μl) was injected into the middle of the upper forelimb with a glass micropipette. A NEPA21 electroporator and a pair of needle electrodes (CUY611P7-4, NEPA GENE) were used for electroporation according to the manufacturer's instructions^3^.

### Muscle cell culture

Newt A1 myoblasts were propagated on gelatin-coated plastic in 65% MEM, 10% fetal bovine serum (FBS), 25% H_2_O, 10 μg ml^−1^ insulin and penicillin/streptomycin^13^. The Amaxa Nucleofector was used for electroporation of A1 myoblasts (Program T30). For myotube purification, confluent myoblasts were cultured in 0.5% serum-containing medium for 4 days to induce myogenic differentiation. The cells were then trypsinized, neutralized in 0.5% serum-containing medium, filtered through 100-μm meshes and the filtrate passed through 35-μm meshes. The myotubes retained on the 35-μm meshes were washed into low-serum medium and plated into a gelatin-coated plate^18^. The cells were left for 2 days after injections before any chemicals were added. The Caspase-Glo 3/7 Assay System (Promega) was used to measure the caspase activity in A1 cells.

C2C12 myoblasts (ATCC CRL-1772) were maintained in the growth medium (20% FBS, DMEM) and transfected with CAG-loxp-mcherry-stop-loxp-h2bYFP plasmid and stably integrated cells were isolated clonally. A MCK-Cre-SV40-zeocin vector was used to establish MCK-Cre stably integrated C2C12 myoblasts. After transfection, myoblasts were cultured in 400 ng ml^−1^ zeocin for 3 weeks and positive clones were pooled together. MCK-Cre myoblasts (2 × 10^4^) and Loxp-h2bYFP myoblasts (4 × 10^4^) were mixed in the proliferation medium and plated into one well of 10% Matrigel-coated 24-well plate. The differentiation medium (DMEM, 2% horse serum) was added the second day. On the fourth day, h2bYFP^+^ myotubes could be easily seen in culture. The medium was changed every other day and EdU was added at 10 μM for 24 h. Primary myoblasts were derived from single muscle fibres isolated from Rosa26:tomato mice^45^ and maintained in growth medium (20% FBS, 10% horse serum, 1% chicken embryo extract, DMEM). MCK-cre myoblasts (1 × 10^4^) and primary myoblasts (2 × 10^4^) were mixed and plated according to the differentiation fusion protocol described above and in a modified protocol described earlier^33^. Three days after differentiation, Tomato^+^ myotubes could be easily seen in culture. Newt and mouse myotubes were treated with STS in the differentiation medium for 2 days and changed to DIDS and Q-VD or ZAVD in the growth medium (supplement with 20 μg ml^−1^ EGF).

### Virus production and transduction

To achieve the P53 knockdown in myotubes specifically, a conditional short hairpin RNA (shRNA) lentivirus plasmid (Addgene 12089 for shP53, Addgene 11578 and sequence 5′-TTCTCCGAACGTGTCACGT-3′ for control) was used^46^. Primary myoblasts were transduced with viral particles at multiplicity of infection 5–10 in polybrene 5–8 μg ml^−1^ for 8 h. Adenovirus CMV-Cre was from Vector Biolabs. A separate differentiation experiment was employed to examine the P53 knockdown efficiency in primary myotubes. Primary myoblasts were transduced with the shRNA knockdown lentivirus (U6:loxp-CMV-GFP-loxp:shRNA) and differentiated into myotubes. Then Adeno cmv-cre was added into the myotube cultures to remove the cmv:GFP cassette and express shRNA. The mRNA and protein levels of P53 were examined.

### Quantitative RT–PCR

The regenerating limbs, including blastema, were collected and snap-frozen in liquid nitrogen. Trizol reagent was used for tissue and cultured cell RNA extractions. The cDNA synthesis was performed with SuperScript III Reverse Transcriptase and quantitative PCR analysis was performed according to ref. 47 and manufacturer's instructions (Life Technologies). The primer sequences were listed in Supplementary Table 2.

### Western blot analysis and immunofluorescence

Total protein was isolated from cells by the RIPA buffer. Protein concentration was determined by the BCA (Pierce). Twenty micrograms of total proteins were resolved using the NuPAGE precast gels (Invitrogen) and transferred to polyvinylidene difluoride membrane. Following blocking with 5% BSA in TBS-T, the membrane was incubated with primary antibodies and secondary antibodies. Immunoreactivity was detected using Amersham ECL Prime (GE). The immunoblots shown represent the results of least three independent experiments (Supplementary Fig. 11). Protein density was measured with ImageJ.

Tissue samples were collected and snap-frozen in isopentane (VWR) liquid nitrogen slurry. For caspase 3 and TUNEL staining, limbs were placed in 4% formaldehyde for 1 h before being frozen. Sections (6–8 μm) were thawed at room temperature and fixed in 4% formaldehyde for 5 min. Sections were blocked with 10% donkey serum in 0.1% Triton-X for 30 min at room temperature. Sections were incubated with a relevant primary antibody overnight at 4 °C and with secondary antibodies for 1 h at room temperature. Antibodies were diluted in blocking buffer and sections were mounted in mounting medium (DakoCytomation) containing 5 μg ml^−1^ 4,6-diamidino-2-phenylindole (Sigma). Myoblasts and myotubes were fixed with 4% formaldehyde or ice-cold methanol for 5 min. Cultured cells were permeablized with 0.5% triton-X for 10 min and blocked with 10% donkey serum, and follow the same staining procedure as tissue sections.

### Microscopy and image processing

Time lapse imaging microscopy was performed on the Leica DMI6000 system. The × 10 images were captured every hour. The green and red fluorescence images were merged and compiled to short videos with the Volocity Demo software. An Axioplan2 microscope (Carl Zeiss) with the Openlab 3.1.7 software (Improvision) was used for fluorescence microscopy analyses. An LSM 700 Meta laser microscope with the LSM 6.0 Image Browser software (Carl Zeiss) was used for confocal analyses. Representative images were selected from at least two independent experiments. For longitudinal sections, 1 in every 20 sections was selected and counted. For transverse sections, 1 in every 15 sections was selected and counted. Blinded counting was performed, as the operator counting the cells was not aware of the type of sample. For counting dedifferentiation frequency, the right (XIAP) and left (control) forelimbs were collected from seven animals at 12 d.p.a. and sectioned transversely. All YFP^+^ nuclei were counted from the regenerate tip to stump until no more YFP^+^ nuclei were found. The number of YFP^+^/MHC^−^ cells in the blastema was normalized to the labelled YFP^+^ myonuclei in the stump (YFP^+^/MHC^+^) before comparing to control.

### Statistical analysis

The animal experiments were not randomized. No statistical method was used to predetermine sample size. No samples were excluded from the statistical analysis. Data were shown as mean±s.e.m. Statistical analyses were performed with Prism 5.0, unpaired two-tailed *t*-test or one-way analysis of variance were applied, unless otherwise stated.

## Additional information

**How to cite this article:** Wang, H. *et al.* Turning terminally differentiated skeletal muscle cells into regenerative progenitors. *Nat. Commun.* 6:7916 doi: 10.1038/ncomms8916 (2015).

## Supplementary Material

Supplementary Figures and Supplementary Tables Supplementary Figures 1-11 and Supplementary Tables 1-2

Supplementary Movie 1 Illustration of the genetic labeling of C2C12 myonuclei.

Supplementary Movie 2 Example of derivation of a dividing mononucleate progeny from C2C12 myotubes.
