# Supplementary material for: Turning terminally differentiated skeletal muscle cells into regenerative progenitors
Source: Nat Commun. 2015 Aug 5;6:7916. doi: 10.1038/ncomms8916 (PMC4765497; doi:10.1038/ncomms8916)
Supplement: Supplementary Figures and Supplementary Tables — Supplementary Figures 1-11 and Supplementary Tables 1-2 [file ncomms8916-s1.pdf]

Supplementary Figure 1

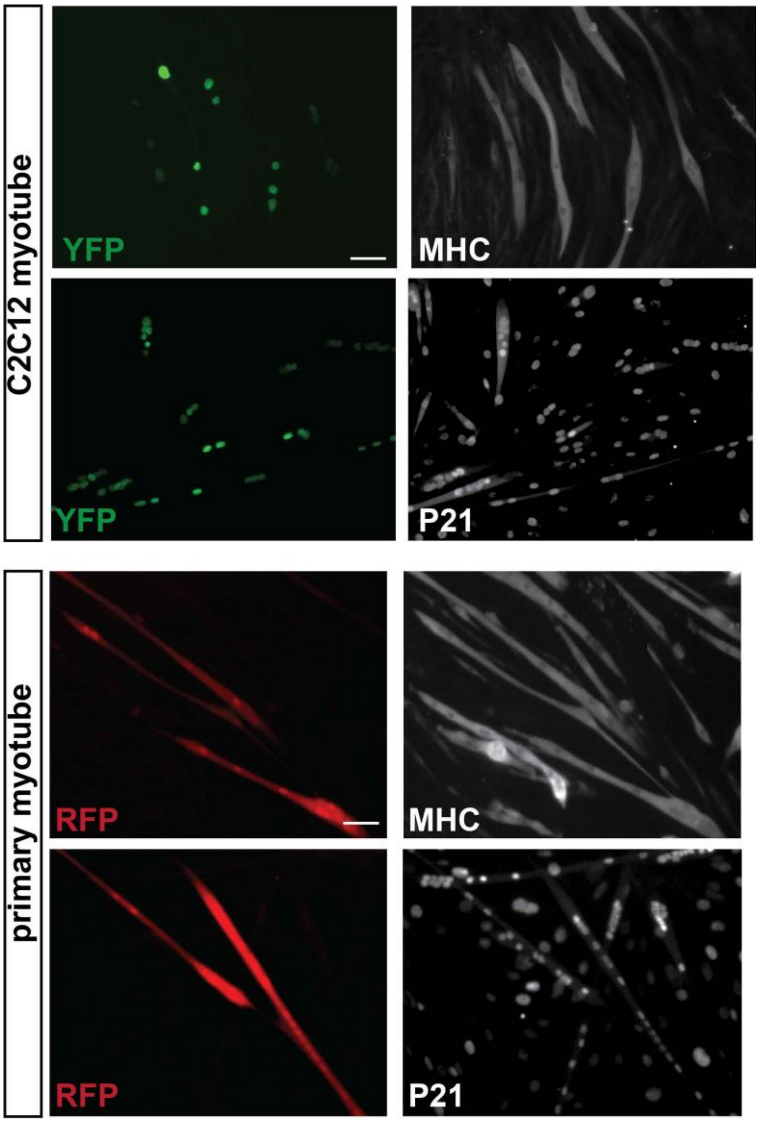

Specificity of the genetic labeling strategy of C2C12 and primary myotubes.

Representative images show labeled myotubes expressing the muscle differentiation marker MHC (C2C12: n = 322, Primary: n = 187) and P21 (C2C12: n = 519, Primary: n = 246). Scale bars, 20μm.

Supplementary Figure 2

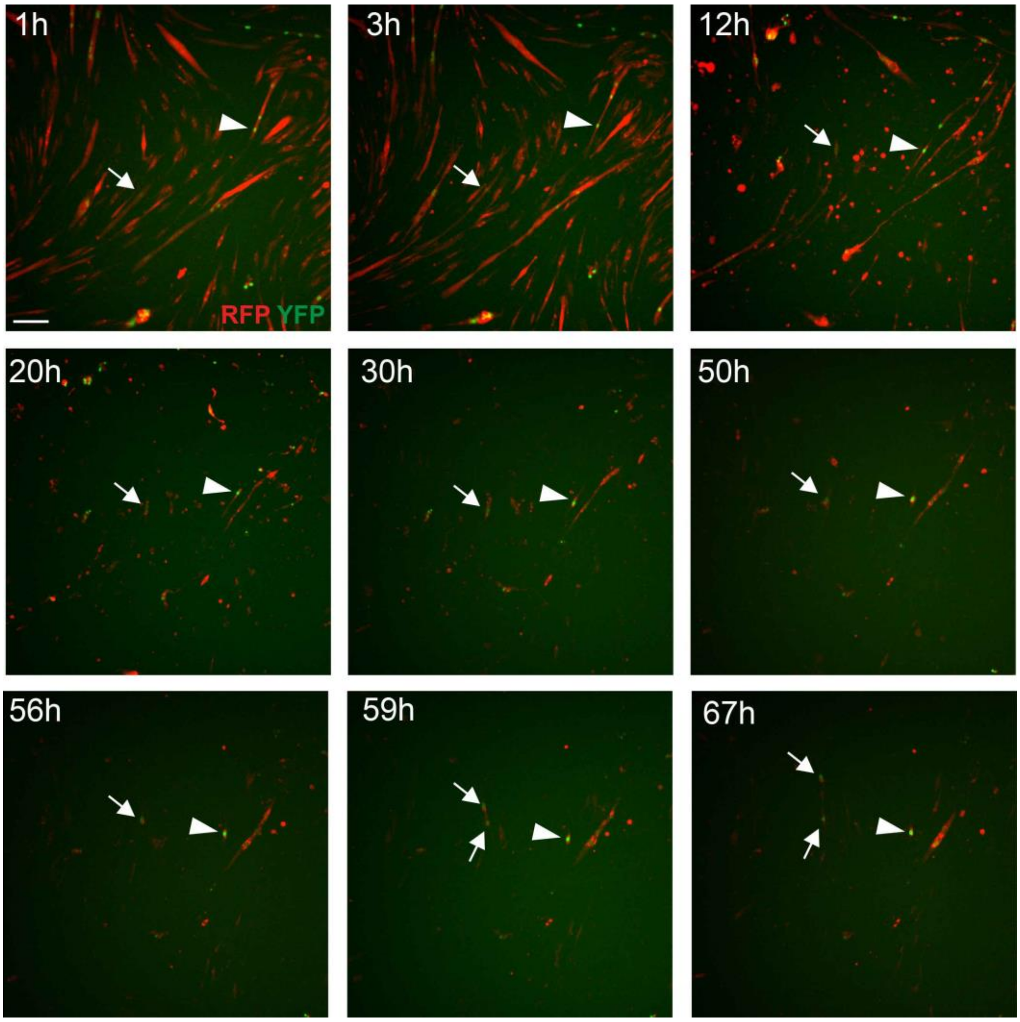

Time-lapse images showing C2C12 myotube fragmentation and subsequent division of a mononucleate progeny.

Arrows point to the sequence of fragmentation and subsequent division. Arrowheads point to a fragmentation event without subsequent division. Relate to Supplementary Movie 2. Scale bar, 20µm.

Supplementary Figure 3

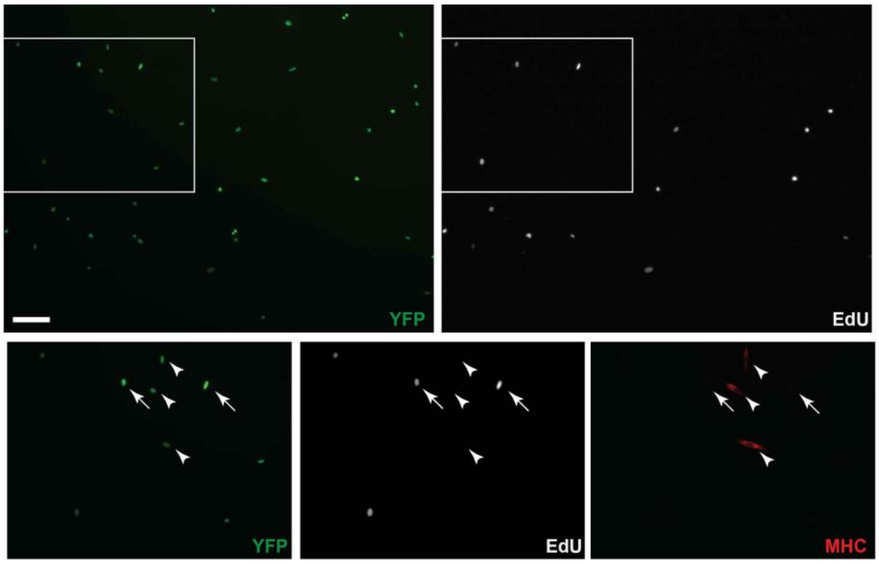

Mononucleate cells derived from C2C12 myotubes a downregulate MHC expression and resume proliferation.

Many YFP^{+} mononucleate incorporate EdU. Only MHC^{-} cells are EdU^{+}. Arrows point to YFP^{+}/EdU^{+}/MHC^{-} cells, arrowheads point to YFP^{+}/EdU^{-}/MHC^{+} cells. Scale bar, 20μm.

Supplementary Figure 4

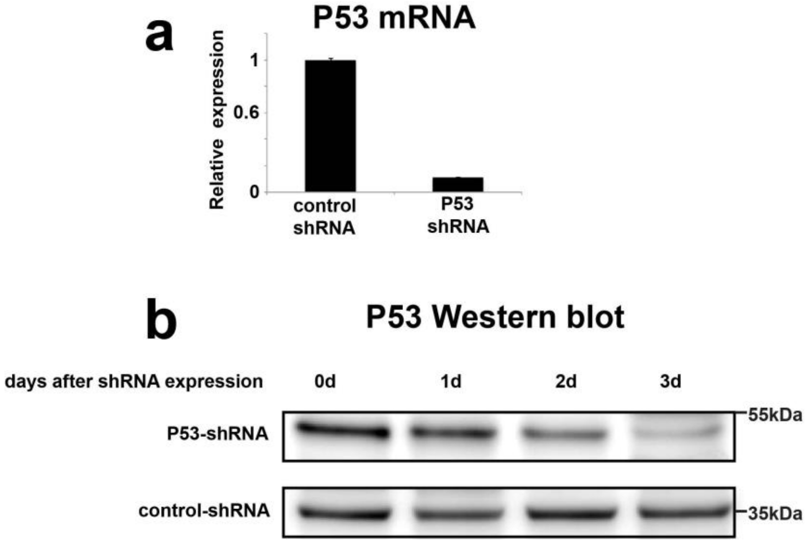

Conditional knockdown of P53 in primary cultures.

(a) Relative expression of p53 mRNA assessed by q-RT-PCR shows efficient knockdown 24h after cre-mediated recombination. (control shRNA: n=4, p53shRNA: n=6).

(b) Western blot analysis showing reduction of p53 protein after p53 knock down.

Supplementary Figure 5

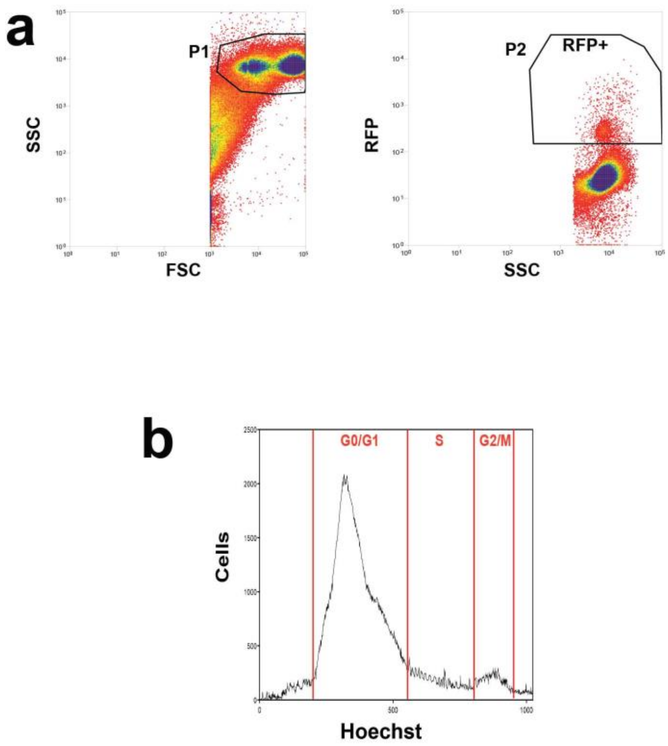

Isolation of the de-differentiated cells by FACS sorting.

(a) Forward scatter and side scatter showing sorting of the RFP^+ cells.

(b) Sorting of the proliferating de-differentiated cells (S and G2/M cells).

Supplementary Figure 6

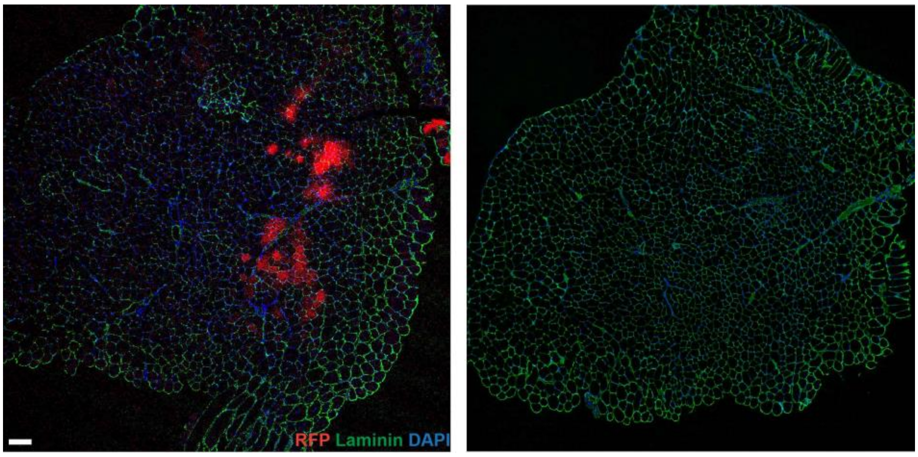

Engrafted de-differentiated cells re-differentiate and fuse to regenerating myofibers *in vivo*.

Representative transverse sections of TA muscle shown in the left panel (related to Fig. 4g) and controls with only secondary antibody in the right panel. Scale bar: 200 μm.

Supplementary Figure 7

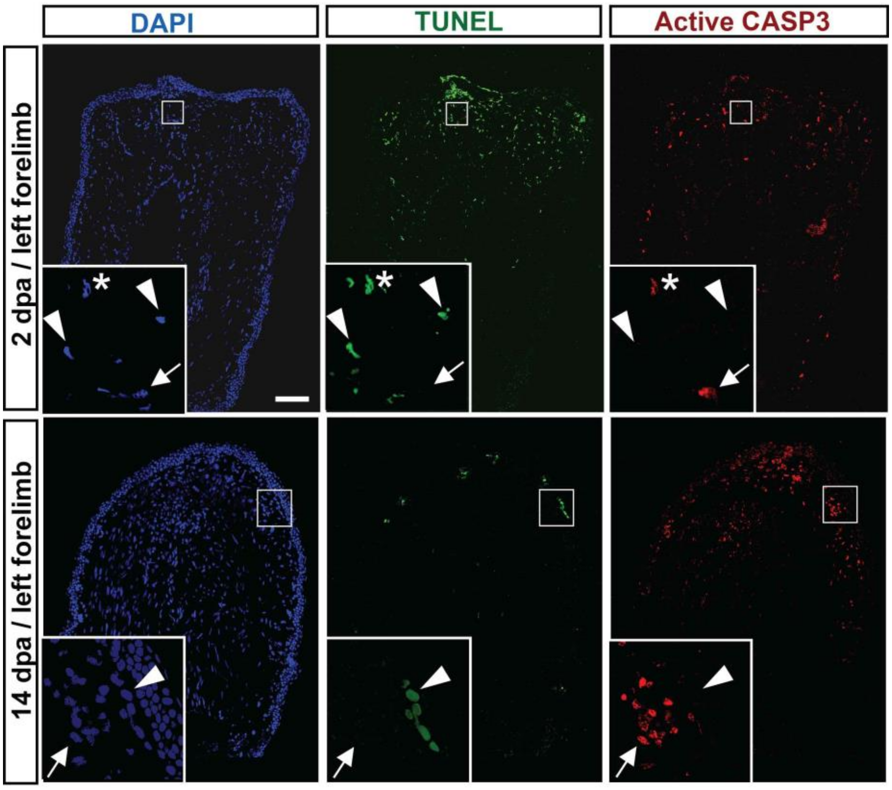

Co-labeling of TUNEL (green) and active-caspase-3 (red) in amputated limbs.

Arrows point to active-caspase3^{+}/TUNEL^{-} cells (7% of cells, 2dpa). Arrowheads point to active-caspase 3^{-}/TUNEL^{+} cells (14% of cells, 2dpa). Asterisks point to active-caspase-3^{+}/TUNEL^{+} cells (3% of cells, 2dpa). Scale bar, 200 µm.

Supplementary Figure 8

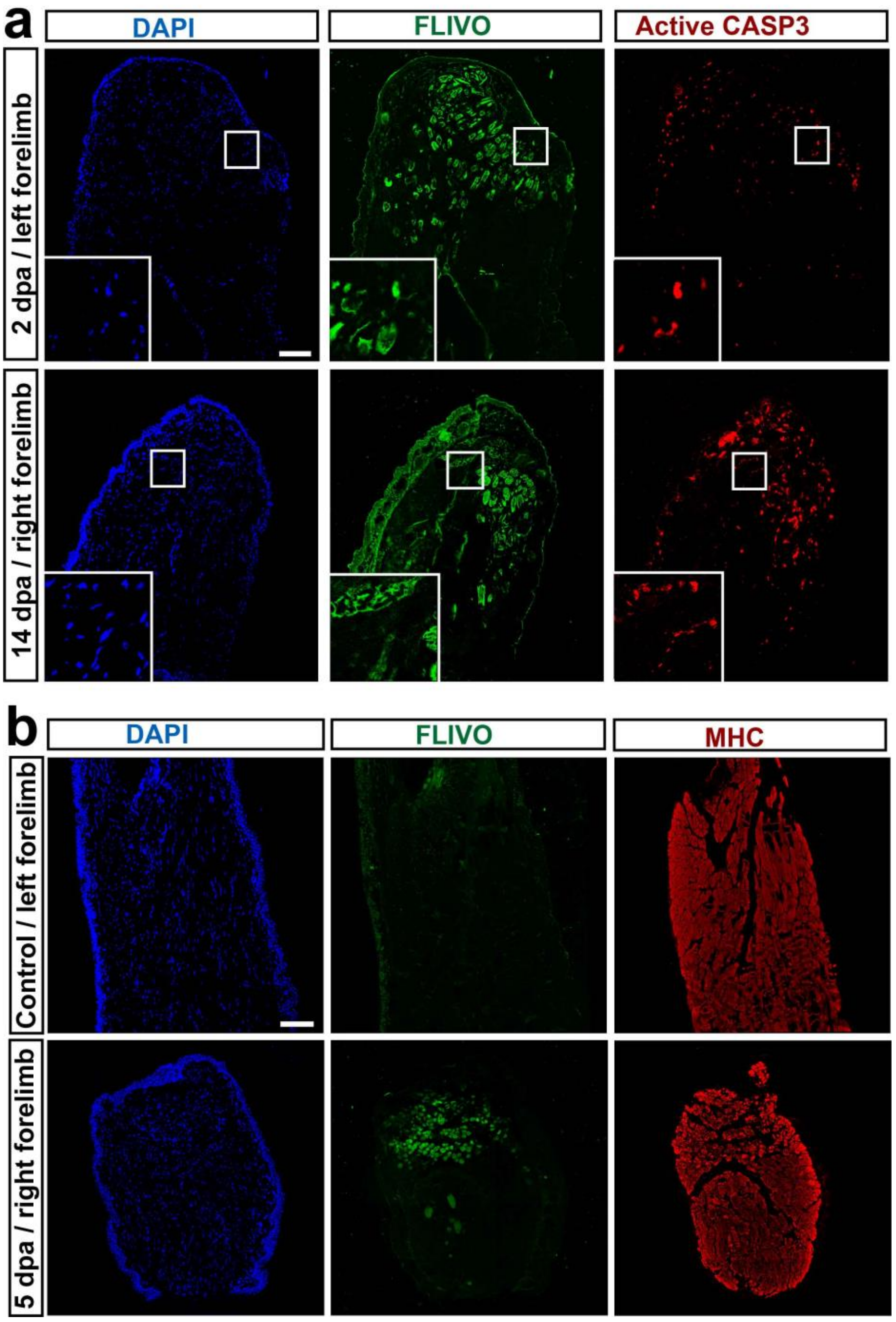

***In vivo* detection of caspase activity with the fluorescent substrate FLIVO.**

(a) Co-labeling of active-caspase-3 (red) and FLIVO (green) in the regenerating limb at different stages of regeneration.

(b) Detection of caspase activity in injured muscle with FLIVO probe. Uninjured limb shows a paucity of FLIVO signal. Scale bars, 200 µm.

Supplementary Figure 9

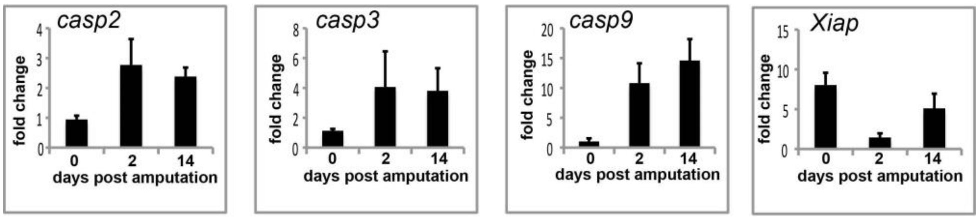

Real-time PCR analysis of PCD related gene products.

The initiator caspases (*CASP2*, *CASP9*), execution caspase (*CASP3*) genes increased significantly after injury. In contrast, *XIAP* expression was reduced. Data are represented as mean ± SEM. n=5 limbs.

Supplementary Figure 10

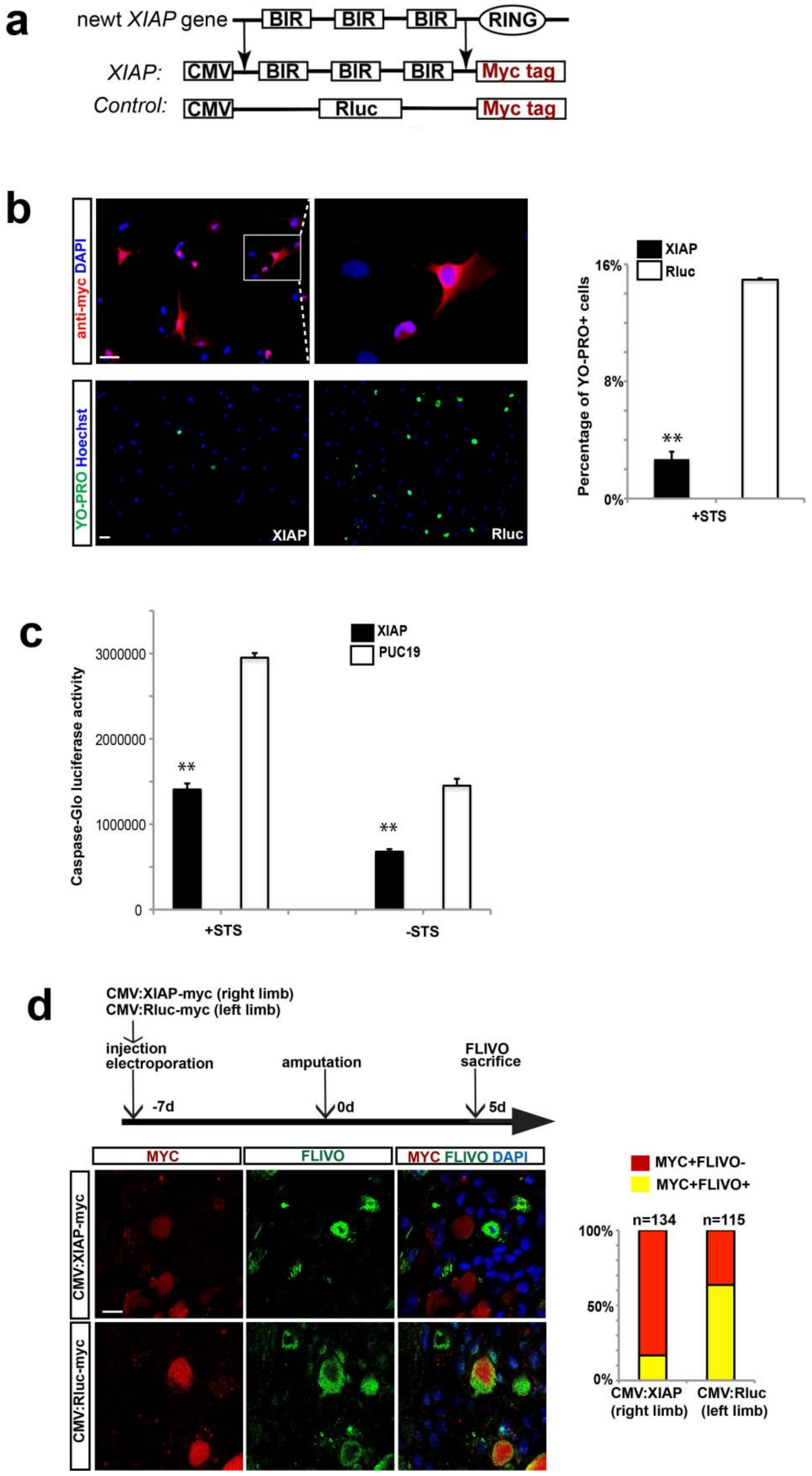

Overexpression of newt XIAP inhibits caspase activity both *in vitro* and *in vivo*.

(a) Schematics of the DNA constructs. The Baculovirus Inhibitor of apoptosis protein Repeat (BIR) domain, but not the RING domain, inhibits efficiently caspases and blocks apoptosis. The coding sequence of three BIR motifs of newt *XIAP* gene was cloned into the pcDNA4 plasmid in reading frame with myc-tag. The Renilla luciferase (*Rluc*) gene was cloned into the same plasmid backbone as control.

(b) XIAP overexpression in cultured newt A1 cells reduces the number of cells undergoing PCD following staurosporine (STS) treatment. Myc-expression (red) is shown in the top panels. YO-PRO (green)-incorporation, which identifies cells undergoing PCD, and Hoechst (blue)-incorporation, which identifies the nuclei are shown in the bottom panel. Quantification of YO-PRO$^+$ cells indicates a reduction in the number cells undergoing PCD upon XIAP-expression. Data are represented as mean ± SEM. N=4 independent experiments with each experiment performed in triplicate. (** $p < 0.01$, Student's t-test). Scale bars, 20 μm.

(c) XIAP overexpression in newt A1 cells inhibits caspase activity compared to control measured with Caspase-Glo luciferase reporter system. Data are represented as mean ± SEM. (n=4, ** $p<0.01$, Student's t-test).

(d) XIAP overexpression in newt limbs inhibits caspase activity in myofibers after injury. Schematic illustration of the experimental strategy (top panel). Transverse sections show myc (red)-expression in transfected myofibers and FLIVO (green)-incorporation indicates caspase activity in myofibers (bottom left panel). XIAP overexpression reduces caspase activity in transfected myofibers (bottom right panel). N is the number of myofibers counted from 4 animals. Scale bar, 20 μm.

Supplementary Figure 11

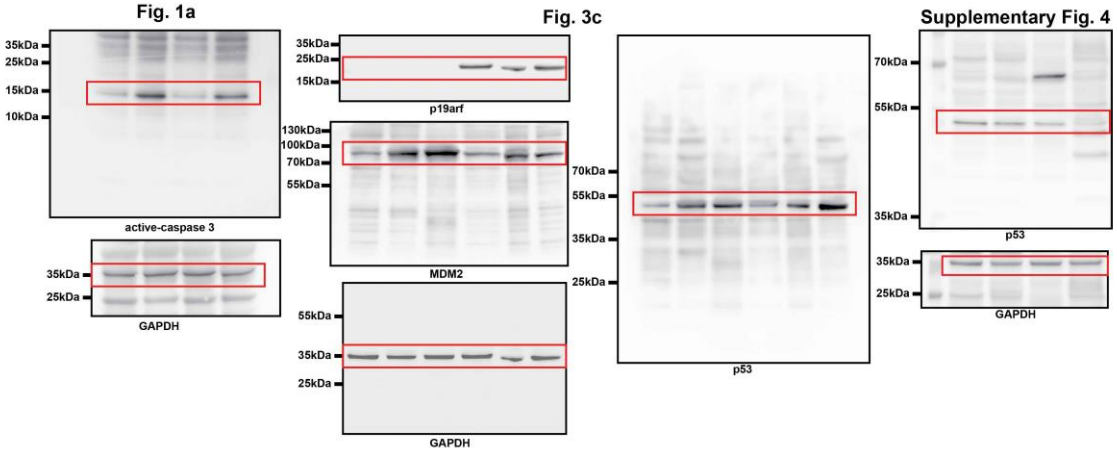

Uncropped scans of western blots

Representative western blots from the experiment in Fig. 1a, Fig. 3c and Supplementary Fig. 4.

Supplementary Table 1. Correlation of nuclear YO-PRO-1 accumulation with myotube fragmentation

**Newt myotubes**	YO-PRO positive	YO-PRO negative
Fragmenting myotubes	35	0
Collapsing myotubes	13	0
Intact myotubes	0	24

Supplementary Table 2. Primers used in this study

Primer name	Sequence (5'-3')
newtCasp2(f)	TGGTGAAGGTGAATGGGATG
newtCasp2(r)	CTCAAAGAAATGGGACGGAC
newtCasp3(f)	AATGGCGGATACAAATGACT
newtCasp3(r)	ATCTTCGCCGTGGCTTAACA
newtCasp9(f)	GAAGTGGCATCAAAAGGGAC
newtCasp9(r)	ATTAACTTCCATGCACTGCC
newtXiap(f)	TTGCTAATTTCCCTGGCAGT
newtXiap(r)	TGTTTGCGAACAGCAGACTC
newtGapdh(f)	CCAAGCGGCAGGTCAGGTCAAC
newtGapdh(r)	TGTGGCGTGACGGCAGAGGTG
mouseP53(f)	CACGTACTCTCCTCCCCTCAAT
mouseP53(r)	AACTGCACAGGGCACGTCTT
mousePax7(f)	GAGTTCGATTAGCCGAGTGC
mousePax7(r)	CGGGTTCTGATTCCACATCT
mousePax3(f)	GCGTCTCTAAGATCCTGTGCAG
mousePax3(r)	GATTTCCCAGCTAAACATGCCCG
mouseMyf5(f)	AACCAGAGACTCCCCAAGGT
mouseMyf5(r)	GCTGGACAAGCAATCCAAGC
mouseMyod(f)	AGTGAATGAGGCCTTCGAGA
mouseMyod(r)	GCATCTGAGTCGCCACTGTA
mouseMyog(f)	GAAGAAAAGGGACTGGGGAC
mouseMyog(r)	GCGCAGGATCTCCACTTTAG
mouseGapdh(f)	TCAACGACCCCTTCATTGAC
mouseGapdh(r)	ATGCAGGGATGATGTTCTGG
